# Tooth Crowns

**Published:** 1886-03

**Authors:** Samuel F. Howland

**Affiliations:** New York


### TOOTH CROWNS.

BY SAMUEL F. HOWLAND, D. D. S., NEW YORK.

Thirty years ago there was but one general method of crowning roots of teeth, and that was by the simple mode of setting a porcelain crown, made for this purpose, on a root, securing the same in place by a wooden dowel. This process of crowning was sightly in its results, but uncleanly, unendurable and unserviceable.

There are many crownless teeth, the bicuspids predominating, yet for this old method crowns were provided for only the six upper front teeth. Bicuspids and molars were forced to remain uncrowned, and were often extracted and their places supplied with teeth on plates.

It once seemed to be the chief business of the dentist to sacrifice teeth; now, it is to save them. It is only within a few years that teeth with devitalized pulps have been successfully treated. Now nearly all teeth and roots may be preserved when it is desirable so to do.

It is well known that natural teeth, which have a normal daily service, are kept in better condition than those which are idle; like other members of the body, work is needful for their health. It is also a fact that many crownless roots which have long been idle and diseased, when properly crowned and given their natural work to do are restored to health and strength.

Within a very few years many new methods of crowning roots have been devised, and the work has received a strong impetus. Among the many new crowns introduced, some are valuable, while others are useless. The best, in some important respects, are deficient. The principal requisites for a tooth crown are naturalness, strength and a secure fastening. With these secured, durability and serviceableness follow.

Recognizing the necessity for something which I had not as yet found, I set about trying to remedy the deficiency, and the result has been a device which I now desire to present to dentists. The crown to which I would call their attention is for a bicuspid, with a bifurcated root, or its equivalent, having two canals, as this permits the fastening by two pins, instead of one; but crowns for this method are made for all the teeth. It is of porcelain, made hollow, and of sufficient size to receive the pins, yet not so large as to impair the strength of the crown. One feature which many will appreciate is, that no metal is to be seen after it is once set. So far as art is able to imitate nature in the construction, it is natural and beautiful, and consequently it will be appreciated by the patient.

The manner of setting is very simple. After the root has been cared for preparatory to this work, it is cut a trifle shorter than the gum with a stump file. A crown of proper size, length and shade is selected, and ground to fit the concave end of the root. The two canals are then enlarged sufficiently to readily receive the threaded pins, made of stiff wire for this purpose. The canals are then partially filled with zinc-phosphate, and with pliers each pin is pushed to its place. To perfect the joint between root and crown, a small quantity of amalgam or warm gutta-percha can be placed on the end of the root and the crown pressed against it. Lastly, the crown is filled with zinc-phosphate and pressed to its place, holding it firmly until the cement has set. If it is desired a gold band can be used. Fit the band on the root, letting it extend a little beyond the end of it, covering so much of the crown as is desired. This is advisable if the root is friable, as it gives additional strength and security to the whole.

I have been inserting crowns in this manner for a number of years. So far they have given excellent satisfaction, and not one has broken so far as is known. It is offered with the hope that it may be of benefit to those who desire a simple and practical method for extending the usefulness of a large class of broken down teeth.

